# Hernie inguinale étranglée compliquée d’ischémie testiculaire sur perméabilité du canal peritonéo-vaginal

**DOI:** 10.11604/pamj.2018.29.76.14132

**Published:** 2018-01-25

**Authors:** Nfally Badji, Chaouch Abdesslem, Niang El Hadji

**Affiliations:** 1 Service de Radiologie Générale du CHU Aristide Le Dantec, Dakar, Sénégal

**Keywords:** Hernie inguinale étranglée, ischémie testiculaire, canal péritonéo-vaginal, Strangulated inguinal hernia, testicular ischemia, peritoneal-vaginal duct

## Abstract

La hernie inguinale de l’enfant est une pathologie fréquente. L’étranglement herniaire est la principale complication de cette pathologie et constitue une véritable urgence diagnostique et thérapeutique. L’imagerie en particulier l’échographie joue un rôle primordial en cas de doute diagnostic surtout lorsque la hernie survient dans un contexte de testicule oscillant car le tableau peut mimer une torsion du cordon spermatique. La réalisation d’une échographie doit être de mise devant un tableau de hernie étranglée dans un contexte de testicule oscillant pour éliminer une ischémie testiculaire associée.

## Abstract (en)

Inguinal hernia is frequent in children. Strangulated hernia is the main complication of this disease and it is a diagnostic and therapeutic emergency. Imaging techniques, in particular Ultrasound, play a key role in case of diagnostic doubt, especially when the hernia occurs in a context of oscillating testicles because this may mimic a torsion of the spermatic cord. Ultrasound should be used in patients with strangulated hernia associated with oscillating testicles in order to eliminate testicular ischemia.

## Introduction

La hernie inguinale de l’enfant est une pathologie fréquente dont l’incidence globale varie entre 0,8 et 13% chez l’enfant, tout âge confondu, et atteint près de 30% chez l’enfant prématuré. Elle appartient aux malformations congénitales par persistance du canal péritonéo-vaginal chez le garçon, et par persistance du canal de Nück chez la fille [1]. L’étranglement herniaire est la principale complication de cette pathologie et constitue une véritable urgence diagnostique et thérapeutique. L’imagerie en particulier l’échographie joue un rôle primordial en cas de doute diagnostic. Nous rapportons un cas d’hernie inguinale étranglée à contenu caecal sur testicule oscillant diagnostiqué à l’échographie au service d’imagerie du CHU Aristide Le Dantec.

## Patient et observation

**Figure 1:**
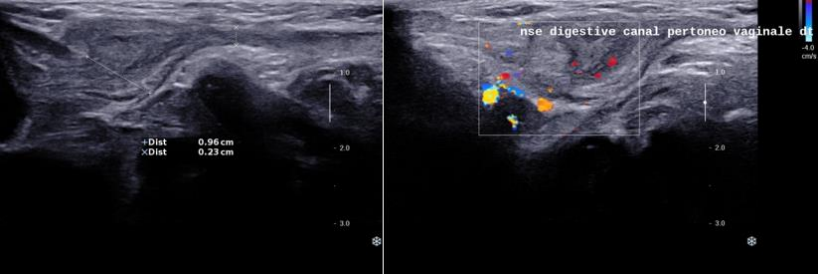
Coupes échographiques longitudinales du canal inguinal: perméabilité du canal péritonéo-vaginal à collet large

**Figure 2:**
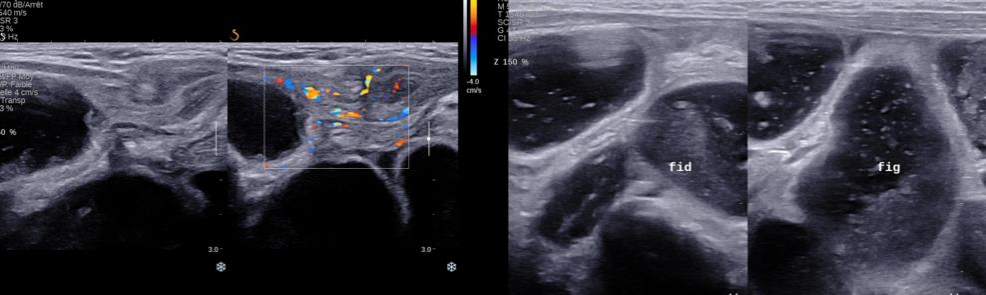
Coupes échographiques longitudinale du canal inguinal et transversale sur l’abdomen, montrant une structure digestive en continuité avec le colon ascendant et une distension intestinale diffuse

**Figure 3:**
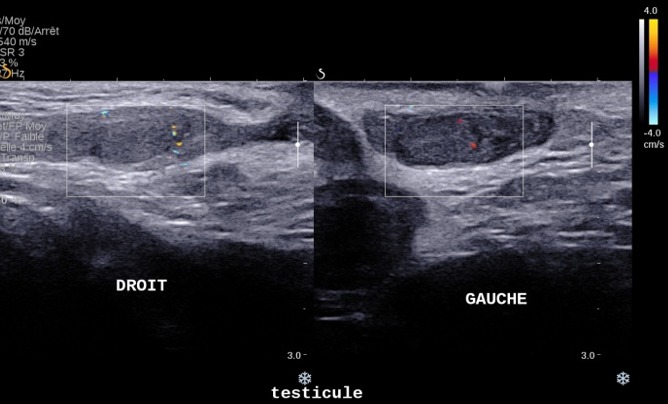
Testicule droit en position inguinale basse d’échostructure discrètement hypoéchogène faiblement vascularisé au doppler énergie: testicule gauche en intrascrotale

**Figure 4:**
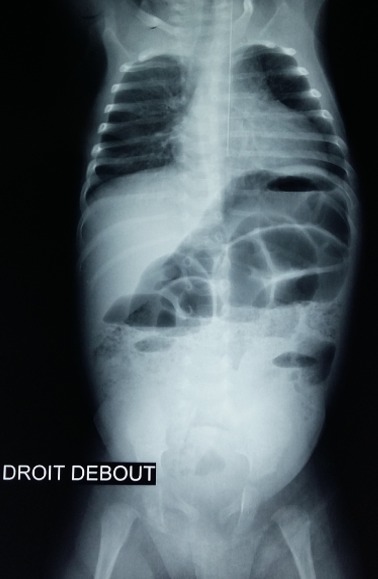
Radiographie de l’abdomen sans préparation en position debout montrant des niveaux hydro-aériques mixtes

**Figure 5:**
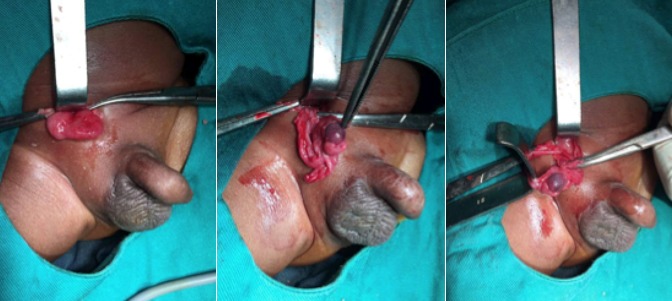
Vues peropératoires montrant le sac herniaire et le testicule ischémié

Nourrisson âgé de deux mois de sexe masculin sans antécédent pathologique particulier se présentait aux urgences pédiatriques pour tuméfaction inguinale droite douloureuse, une torsion du cordon spermatique à été suspecté devant la constatation clinique d’une ascension du testicule homolatérale et une échographie Doppler testiculaire à été demandée. Une échographie réalisée en urgence a permis de mettre en évidence une persistance totale du canal péritonéo-vaginal (CPV) droit longueur avec un diamètre mesuré à 9 mm avec incarcération d’une anse digestive, le coecum, au niveau de sa portion proximale (Figure 1) non réductible à la pression par la sonde avec une importante distension colique et grélique diffuse d’amont. La paroi caecale était épaissie, non dédifférenciée et hyperhémiée en Doppler couleur (Figure 2). Le testicule homolatéral était en situation inguinale basse de taille normale hypoéchogène homogène faiblement vascularisé au Doppler couleur sans image de torsion du cordon spermatique (Figure 3). Le testicule droit est en situation intrascrotale d’aspect normal. Le diagnostic de hernie inguinale étranglée à contenu caecal compliquée de souffrance testiculaire homolatérale était retenu. Une radiographie de l’abdomen sans préparation montrait des niveaux hydro-aériques confirmant l’occlusion intestinale (Figure 4). Le diagnostic a été confirmé par la chirurgie (Figure 5). Les suites opératoires étaient simples.

## Discussion

La hernie inguinale du nourrisson contrairement à celle de l’adulte est congénitale (hernie indirecte, et oblique externe) due à l’absence de régression du canal péritonéo-vaginal (CPV) chez le nourrisson de sexe masculin. Il s’agit d’une pathologie fréquente du nourrisson et de l’enfant. L’étranglement herniaire est la principale complication et constitue une véritable urgence diagnostique et thérapeutique.

Pour mieux comprendre le mécanisme physiopathologique des diverses pathologies rencontrées en cas de persistance du CPV nous nous sommes proposés de faire un rappel embryologique du développement de la région inguino-scrotale. La formation de la région inguinale débute au 3^ème^ mois de gestation par une évagination en doigt de gant du péritoine appelé, processus vaginalis ou processus vaginal (PV), et se trouve en position ventrale au gubernaculum. Le PV émerge à travers la paroi abdominale le long d´un chemin formé par le gubernaculum, et chez le fœtus mâle, il passe dans le scrotum en développement. Les extensions des couches de la paroi abdominale accompagnant le PV forment les murs du canal inguinal et aussi les revêtements du cordon spermatique et des testicules. L´ouverture du fascia transversalis produite par le PV forme l´anneau inguinal profond, et l´ouverture triangulaire dans l’aponévrose de l´oblique externe à partir de laquelle le PV émerge forme l´anneau inguinal superficiel.

Chez le garçon, ce diverticule est appelé canal péritonéo-vaginal, il suit le trajet du gubernaculum testis qui permet au testicule de migrer depuis la crête uro-génitale jusqu’au scrotum. Entre le 7^ème^ et le 8^ème^ mois de grossesse, dirigé par les fibres centrales du gubernaculum testis, le testicule passe l´anneau externe et descend dans le scrotum. Entre le 8^ème^ et le 9^ème^ mois, le processus vaginal régresse. Au niveau du canal inguinal, il se ferme complètement et ne laisse qu´un mince cordon fibreux: le ligament de Cloquet. Sa partie distale ne subit aucun changement et forme la vaginale du testicule [2,3]. Chez la fille, le processus vaginal est appelé canal de Nück. Il suit le trajet du ligament rond qui s´étend depuis l´annexe jusqu´à la grande lèvre [4], ce canal se résorbe à la fin de la grossesse ou au cours des premiers mois de la vie.

Ainsi, quel que soit le sexe, la pathologie congénitale de la région inguinale est la conséquence de la non-régression du processus vaginal. Les anomalies de fermeture du processus vaginal sont très fréquentes puisque près de 80% des nouveau-nés auraient un CPV ou un canal de Nück perméable, proportion qui avoisine les 100% chez le prématuré. Certes le diagnostic clinique d’une hernie reste avant tout clinique, mais ce dernier ne peut préjuger de la nature du contenu hernié dominé par le tube digestif en particulier les anses grêles mais aussi de l’état de la vascularisation de ce dernier. Selon Galinier P et al [3] dans l’absolu, les examens radiologiques ont peu d’intérêt, dans le diagnostic d’une hernie.

Cependant l’abdomen sans préparation (cliché réalisé debout) peut, dans le cas d’une hernie étranglée, mettre en évidence une image de niveau hydro-aérique se projetant dans la région inguinale. L’échographie réalisée à l’aide d’un appareil haute résolution peut, dans certaines situations, apporter une aide au diagnostic, notamment dans le cas d’une petite hernie funiculaire étranglée associée à une hydrocèle, en confirmant la présence d’une anse incarcérée dans la partie haute du canal inguinal. En plus l’échographie permettra de faire le diagnostic différentiel des tuméfactions inguinales. Le kyste du cordon et l’hydrocèle vaginal communicant ou non constituent les principaux diagnostiques différentiels et sont dus à une régression incomplète du CPV [5].

Elle occupe une place capitale dans la prise en charge des hernies inguinales de l’enfant d’autant plus que ces dernières surviennent dans un contexte de testicule oscillant. Une torsion du cordon spermatique peut mimer le tableau et ce d’autant plus que la bourse homolatérale est déshabitée et c’était le cas de notre patient. La réalisation de l’échographie avait permis de redresser le diagnostic en mettant en évidence une anse digestive, le coecum, incarcérée au niveau du CPV non réductible à la pression par la sonde avec une importante distension colique et grélique diffuse d’amont et un testicule homolatérale en situation inguinale basse de taille normale hypoéchogène homogène faiblement vascularisé au Doppler couleur. Le diagnostic de torsion du cordon spermatique avait été éliminée. La hernie inguinale à contenu caecale est une forme rare. Ait Hamou Laila [6], dans son étude sur 52 cas de hernies étranglées 03 avaient un contenu caecal dont une était compliquée de souffrance testiculaire homolatérale. Rantomolo et al [7] ont trouvé 03 cas. La physiopathotologie n’étant pas bien précise, on aurait incriminé plusieurs facteurs favorisants tels que la largeur du collet, un défaut d’accolement du coecum et / ou une anomalie de longueur du colon droit. Une incarcération du coecum pourrait expliquer aisément une compression des vaisseaux du cordon spermatique qui serait à l’origine d’une ischémie testiculaire et simulerait un tableau de torsion spermatique comme c’est le cas de notre patient.

Le diagnostic de hernie inguinale est suspecté, devant l’apparition d’une tuméfaction inguinale ou inguino-scrotale. Une exploration échographique de la région inguinale, est nécessaire car permettra de faire le diagnostic positif en montrant le collet du sac herniaire ainsi que son contenu (graisse mésentérique, intestin grêle, colon, ovaire, appendice), de préciser le caractère étranglé par des manouvres dynamiques de pression avec la sonde échographique [8]. Les éléments échographiques en faveur d’une hernie étranglée sont la présence de signe d’ischémie (épanchement liquidien au niveau du sac herniaire, épaississement pariétal, absence de péristaltisme intestinal, hyperhémie au Doppler couleur présente au début et l’absence de flux sanguin au stade ultimes). La présence d’air dans le sac herniaire ou une pneumatose intestinale ou la perforation intestinale signe la nécrose [9, 10]. Elle permet d’apprécier également le retentissement de la hernie sur le testicule homolatéral, de faire le diagnostic différentiel minimisant ainsi les incidents per-opératoires en cas d’anomalie congénitale associée tel qu’un testicule oscillant.

## Conclusion

L’imagerie en particulier l’échographie joue un rôle primordial en cas de doute diagnostic surtout lorsque la hernie survient dans un contexte de testicule oscillant car le tableau peut mimer une torsion du cordon spermatique. La réalisation d’une échographie doit être de mise devant un tableau de hernie étranglée à contenu caecal en particulier dans un contexte de testicule oscillant pour éliminer une ischémie testiculaire associée.

## Conflits d’intérêts

Les auteurs ne déclarent aucun conflit d'intérêts.
